# Role of Steroids on the Membrane Binding Ability of Fatty Acid Amide Hydrolase

**DOI:** 10.1089/can.2018.0051

**Published:** 2019-03-13

**Authors:** Annalaura Sabatucci, Monica Simonetti, Daniel Tortolani, Clotilde B. Angelucci, Enrico Dainese, Mauro Maccarrone

**Affiliations:** ^1^ Faculty of Bioscience, and Technology for Food Agriculture and Environment, University of Teramo, Teramo, Italy.; ^2^ Faculty of Veterinary Medicine, University of Teramo, Teramo, Italy.; ^3^ European Center for Brain Research (CERC)/Santa Lucia Foundation, Rome, Italy.; ^4^ Department of Medicine, Campus Bio-Medico University of Rome, Rome, Italy.

**Keywords:** docking, FAAH, FRET, membrane binding, pregnenolone, steroids

## Abstract

**Background:** Fatty acid amide hydrolase (FAAH) is a membrane-bound homodimeric enzyme that gets in contact with a lipophilic substrate in the lipid bilayer, and then cleaves it into water soluble products. FAAH plays a critical role in modulating *in vivo* content and biological activity of endocannabinoids (eCBs), and its function is affected by membrane lipids. Increasing evidence suggests that also steroids can modulate endocannabinoid signaling, both in the central nervous system and at the periphery.

**Methods:** In this study, we interrogated the effect of six steroids with relevant biological activity (testosterone, hydrocortisone, estradiol, pregnenolone, progesterone, and cortisone) on the membrane binding ability of rat FAAH. The experimental data analysis obtained by Fluorescence Resonance Energy Transfer Spectroscopy was paralleled by computational docking analysis.

**Results:** Our data revealed distinct effects of the different steroids on the interaction of rat FAAH with model membranes. Among them, pregnenolone was found to be the most effective in raising rat FAAH affinity for model membranes. A possible binding pocket for steroid molecules was identified by docking analysis in the membrane-embedded region of the enzyme; such a pocket could account for the observed increase of the membrane affinity in the presence of the tested molecules.

**Conclusions:** Overall, the results point to steroids as new regulators of FAAH interaction with membranes, which may impact the biological activity of eCBs.

## Introduction

Endocannabinoids (eCBs) are bioactive lipids that are mainly produced “on demand” (i.e., when and where needed upon physiological and/or pathological stimuli) from membrane lipid precursors, through multiple biosynthetic pathways. Many research efforts have shed light on the impact of eCBs on human health and disease, identifying an ensemble of proteins that bind, synthesize, and degrade them, altogether forming the endocannabinoid system (ECS).^1–6^

*N*-arachidonoylethanolamine (anandamide, AEA) and 2-arachidonoylglycerol (2-AG), the most studied eCBs, show different affinities for type-1 (CB_1_) and type-2 (CB_2_) cannabinoid receptors,^7^ which are two well-characterized 7-transmembrane helices G protein-coupled receptors. Accumulated evidence suggests the occurrence of other membrane proteins and receptors that are targets for eCBs, among which are the transient receptor potential vanilloid 1 ion channel^8^ and the orphan receptor GPR55.^9^ Metabolism of AEA and 2-AG occurs through distinct pathways that have been described in detail.^10^ Overall, there is consensus that the *in vivo* biological activity of eCBs is under a metabolic control. In particular, fatty acid amide hydrolase (FAAH), which breaks the amide bond of AEA (and to a lesser extent the ester bond of 2-AG) to release arachidonic acid, has been recognized as a key regulator of endocannabinoid signaling *in vivo*.^11^ Indeed, FAAH belongs to the large “amidase signature” (AS) family of hydrolytic enzymes that contain a conserved stretch of ∼130 residues known as the AS sequence. Much alike the other members of the AS family, FAAH possesses a unique highly conserved Ser-Ser-Lys catalytic triad used for amide hydrolysis.^12^

The first obtained crystal structure of a recombinant rat FAAH (rFAAH) showed a dimeric functional unit that corresponds to the membrane-bound form.^13^ The X-ray structure of rFAAH in complex with the nonsteroidal anti-inflammatory drug (NSAID) carprofen revealed that the latter inhibits enzyme activity by binding to a region located at the entrance of the membrane access (MA) port, thus preventing substrates to reach the active site.^14^

Membrane lipids could also modulate structure, functional activity, and subcellular localization of FAAH. Indeed, the FAAH dimer is stabilized by the lipid bilayer and shows a higher membrane-binding affinity and enzymatic activity within membranes containing both cholesterol and the FAAH substrate, AEA.^15^ In addition, the colocalization of cholesterol, AEA, and FAAH in intact cells supports a mechanism by which cholesterol can increase substrate accessibility to the active site.^15^ Of note, FAAH interacts mainly with one leaflet of the membrane bilayer with a region belonging to the MA port,^13^ which appears the only access for AEA to the active site. Such an access can be opened by cholesterol, with subsequent increase of AEA accessibility.^15^

Functional interactions between eCBs and steroids have emerged both centrally and peripherally.^16^ Steroids and their secondary metabolites are a group of cholesterol-derived lipophilic compounds that play important roles in the physiology of living organisms. Steroidogenic enzymes are present in numerous tissues such as adrenal gland, testis, ovary, brain, placenta, and adipose tissue. They consist of diverse cytochrome P450 (CYP450) enzymes, hydroxysteroid dehydrogenases, and steroid reductases,^17^ responsible for the biosynthesis of glucocorticoids, mineralocorticoids, progestins, androgens, and estrogens from cholesterol. *De novo* synthesis of all steroid hormones starts with the conversion of cholesterol to pregnenolone by a cholesterol side chain cleaving enzyme expressed only in steroidogenic tissues.^18^ Subsequently, pregnenolone is converted to progesterone by 3β-hydroxysteroid dehydrogenase, one of several non-CYP450 enzymes involved in steroidogenesis, which is found in both mitochondria and smooth endoplasmic reticulum (ER). Available data suggest that steroids can modulate the eCB tone, through genomic or nongenomic regulation, and that eCBs can complement the biological activity of steroids.^19^ In this context, an increasing debate concerns the tissue- and species-specificity of the eCB–steroid interface, and the possibility that eCBs can modulate steroid metabolism. As an example, an important role for eCBs has been suggested in the regulation of sex hormone-dependent tumors and metastasis.^20^

Moreover, the crosstalk between steroids and eCBs might be a key to interpret molecular events responsible for reproductive function, and, in particular, its immune regulation,^21,22^ as well as for drug addiction and alcohol dependence.^16^

Against this background, in this study we sought to ascertain whether steroid hormones can modulate the membrane affinity of FAAH. To this aim, the possible effect of six steroids with a relevant biological activity was interrogated on the binding of rFAAH to model membranes, by using fluorescence resonance energy transfer (FRET) spectroscopy, and by *in silico* analysis. In particular, we chose four steroids with a C21 pregnane skeleton (cortisone, progesterone, hydrocortisone, and their precursor pregnenolone), one (testosterone) with an androstene skeleton (C19), and another one (estradiol) with an estrane skeleton (C18). Docking analysis showed a hydrophobic binding pocket of the enzyme with different interactions for the investigated steroids, which could account for their different contributions to the enzyme binding affinity to membranes obtained by FRET. Taken together, these results demonstrate an unprecedented molecular interaction of steroids with rFAAH, which appears able to modulate the membrane binding properties of the enzyme.

## Results

### Determination of membrane binding properties of rFAAH in the presence of steroids by FRET

**FIG. 1.**
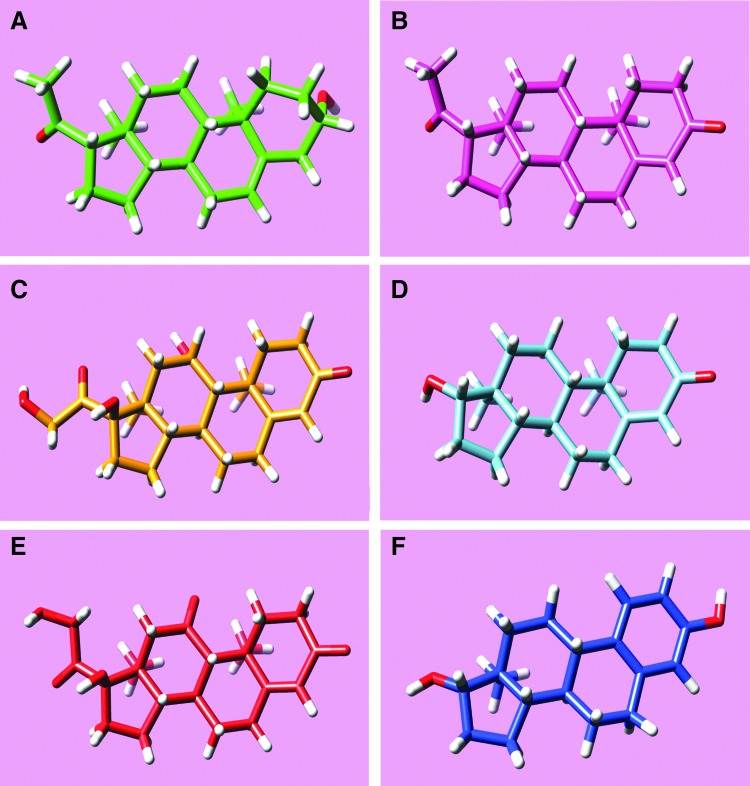
Chemical 3D structures of tested steroids added to POPC liposomes for FAAH–membrane interaction determination by FRET: **(A)** pregnenolone (green), **(B)** progesterone (pink), **(C)** hydrocortisone (yellow), **(D)** testosterone (cyan), **(E)** cortisone (red), and **(F)** estradiol (blu). 3D, three-dimensional; FAAH, fatty acid amide hydrolase; FRET, fluorescence resonance energy transfer; POPC, 1-palmitoyl-2-oleoyl-*sn*-glycero-3-phosphocholine.

The role of steroids in the membrane binding properties of rFAAH was investigated by measuring FRET of recombinant rFAAH with model membranes consisting of large unilamellar vesicles (LUVs), made of the phospholipid 1-palmitoyl-2-oleoyl-*sn*-glycero-3-phosphocholine (POPC) and each of six different steroids: hydrocortisone, progesterone, pregnenolone, testosterone, estradiol, and cortisone (Fig. 1).

For each molecule, two different lipid/steroid molar ratios were analyzed. At the lower molar ratio (300:1), steroid concentration was 1 μM, that is, about the same concentration as the protein. At the higher lipid–steroid molar ratio (30,000:1), the steroid concentration was about 10 nM, that is, close to a physiological value.

As a control, the half saturation binding concentration (L_1/2_) of rFAAH for pure POPC liposomes was measured. The obtained value of L_1/2_=60±4 μM was in good accordance with a previously reported value of 67±10 μM.^15^

**FIG. 2.**
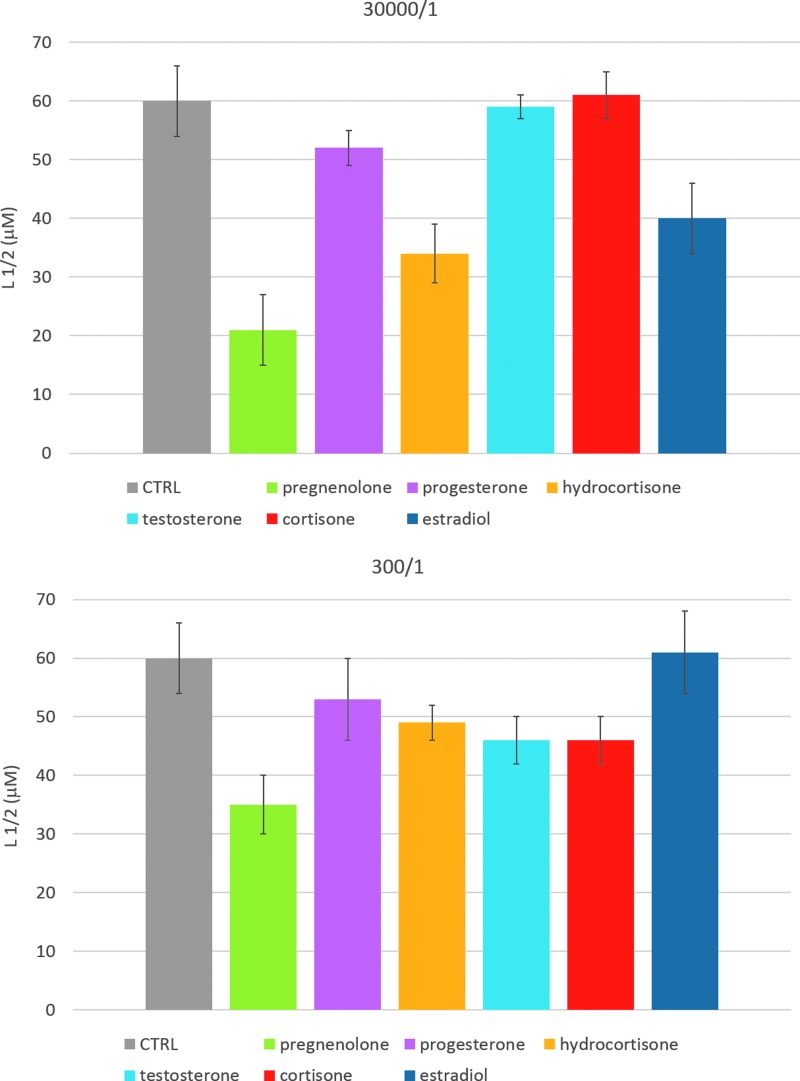
Half saturation concentration values (L_1/2_) for the binding of purified rFAAH to POPC liposome solutions containing steroid molecules at a phospholipid–steroid molar ratio of 30,000/1 (upper panel) and 300/1 (lower panel). Control values are referred to the binding of rFAAH to pure POPC membranes. Statistical analysis is reported in Table 1. rFAAH, recombinant rat FAAH.

As shown in Figure 2, the presence of steroids increased the binding of rFAAH to liposomes at both analyzed lipid–steroid ratios. Statistical analysis of the data confirmed the significance of the calculated parameters (Table 1). In particular, testosterone and cortisone seemed to be more effective at the higher ratio, whereas hydrocortisone, estradiol, and pregnenolone appeared more effective in raising rFAAH membrane affinity already at low concentrations.

**Table 1.** Half Saturation Binding Lipid Concentration for Recombinant Rat Fatty Acid Amide Hydrolase/POPC Interaction in the Presence of Steroids

	30,000/1 POPC–steroid ratio		300/1 POPC–steroid ratio	
	L_1/2_ (μM)	L_1/2_ vs. control (%)	L_1/2_ (μM)	L_1/2_ vs. control (%)
Control	60±6	100.00	60±6	100.00
Pregnenolone	21±2	35.00^***^	35±5	58.33^***^
Progesterone	52±4	86.67	53±7	88.33
Hydrocortisone	34±3	56.67^***^	49±3	81.67^*^
Testosterone	59±6	98.33	46±4	76.67^*^
Cortisone	61±6	101.67	46±4	76.67^*^
Estradiol	40±5	66.67^*^	61±7	101.67

^***^ Denotes *p* value < 0.001; ^*^0.01< *p* value < 0.05.

POPC, 1-palmitoyl-2-oleoyl-*sn*-glycero-3-phosphocholine.

Remarkably, pregnenolone was the most effective enhancer of rFAAH membrane binding affinity, both at low and high lipid–steroid molar ratios.

### Docking analysis

FRET measurements showed that steroids can affect the membrane affinity of FAAH, thus we performed a ligand-protein docking analysis in search for possible binding pockets for these molecules in the membrane-exposed region of rFAAH surface. The analysis was performed on three different rFAAH crystal structures: 3QK5.pdb, 4DO3.pdb, and 2VYA.pdb.

The first (3QK5.pdb) is the structure of rat ΔTM FAAH at a resolution of 2.2Å in the presence of a noncovalent pyrrolopyridine inhibitor (3-{(3R)-1-[4-(1-benzothiophen-2-yl)pyrimidin-2-yl]piperidin-3-yl}-2-methyl-1H-pyrrolo[2,3-b]pyridin-1-yl)acetonitrile. This aryl-pyrimidine makes van der Waals contacts with several hydrophobic residues including F432, M436, L433, W531, T488, and I491.^23^ The second (4DO3.pdb) is the already mentioned X-ray structure of rFAAH in complex with the NSAID carprofen at a resolution of 2.25Å.^14^ The third (2VYA.pdb) is the structure of an rFAAH variant in the presence of the PF-450 inhibitor bound in the active site at a resolution of 2.75Å. In the latter protein, by site-directed mutagenesis, the active site is interconverted to the human one, while the overall fold is essentially identical to the one of rFAAH.^24^

**FIG. 3.**
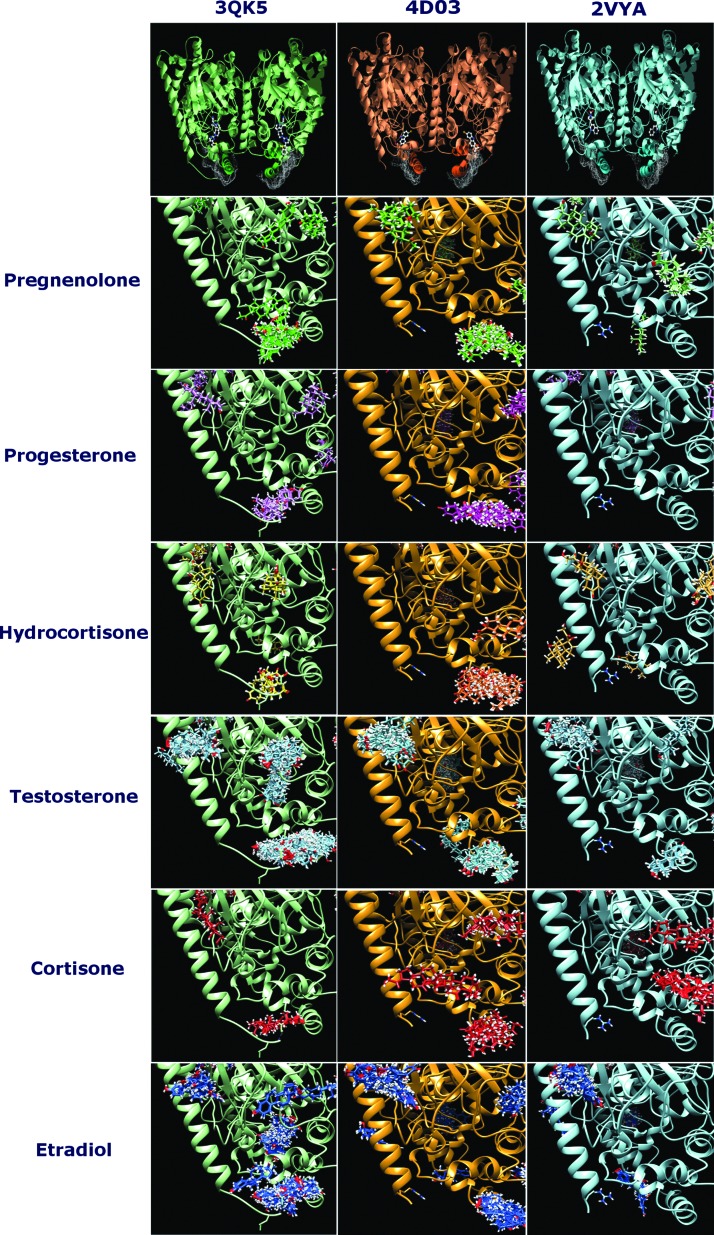
First row: Dimeric structural units of the rFAAH crystal structures 3QK5.pdb (green), 4DO3.pdb (orange), and 2VYA.pdb (cyan). Evidenced in each structure the hydrophobic intramembrane patches as white surfaces. Ball and stick are the cocrystallized inhibitor molecules, respectively, in 3QK5.pdb, (3-{(3R)-1-[4-(1-benzothiophen-2-yl)pyrimidin-2-yl]piperidin-3-yl}-2-methyl-1H-pyrrolo[2,3-b]pyridin-1-yl)acetonitrile; in 4DO3.pdb the NSAID caprofen and in 2VYA.pdb PF-450. From row 2 to 7, the BMs in the area near the hydrophobic intramembrane patches of the three crystal structures are drawn for the investigated six steroids (colors as in Fig. 1). BMs, binding modes.

As shown in Figure 3, our analysis revealed that in the three crystal structures, steroids did not bind to the same site as the inhibitors, rather they were more likely found in a binding pocket located in the hydrophobic membrane-embedded region of the protein, comprising the two helices named α-18 (S412-P424) and α-19 (R428-S435). This region is just below the dynamic paddle formed by the residues Y432 and W531, both essential for substrate specificity^25^ of rFAAH, and is almost identical in the three crystal structures (Fig. 3A). Interestingly, this seems the only region of the protein that could account for a contribution in membrane–protein interaction. The other potential binding pockets identified for steroids lie either in the cytoplasm-exposed surface of the protein, far from the membrane, or in the forbidden intersubunit contact surface.

**FIG. 4.**
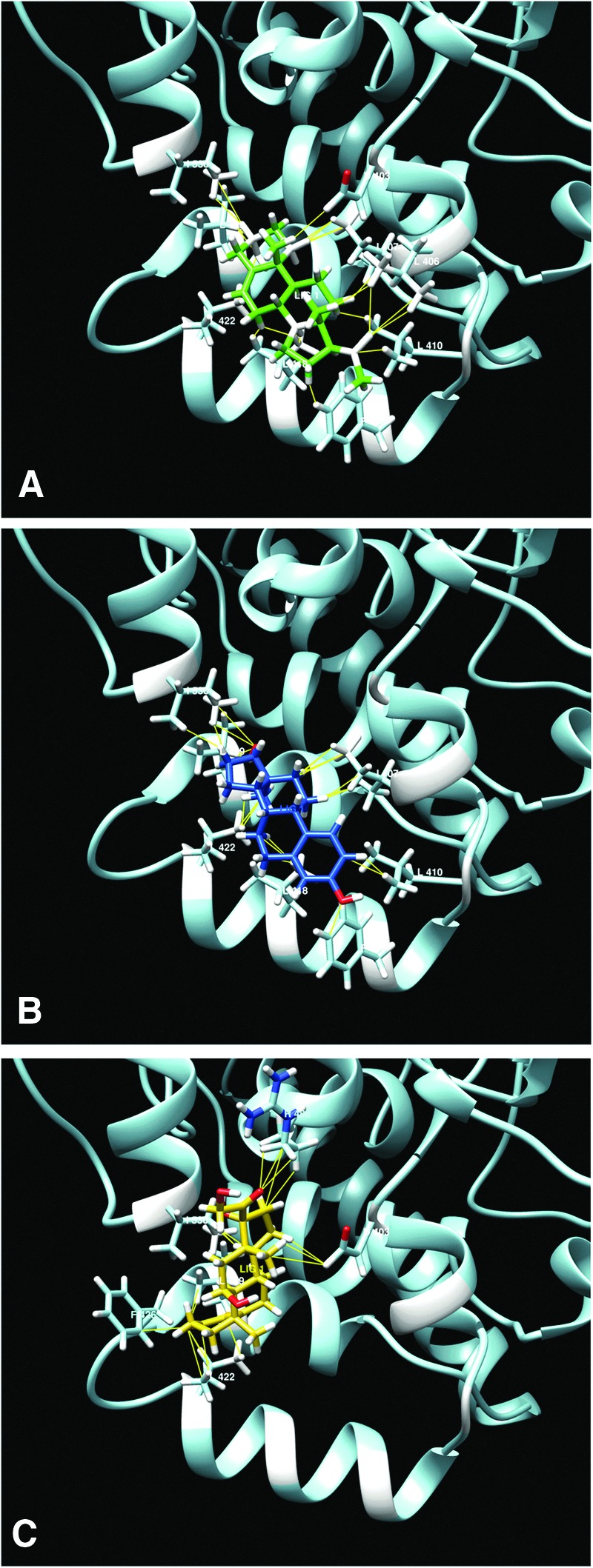
**(A)** Pregnenolone (green), **(B)** estradiol (blue), and **(C)** hydrocortisone (orange) best BMs for rFAAH crystal structure 2VYA.pdb in the pocket situated in the hydrophobic region by the two helices α-18 and α-19. Highlighted are the residues involved in the binding. F432, crucial for substrate access, is not directly involved in the binding.

In particular, among the six steroids analyzed, pregnenolone, estradiol, and hydrocortisone showed energetically favored clusters for this pocket in the structure 2VYA.pdb. In Figure 4, the best binding modes (BMs) of these three molecules within the identified pocket are reported. Interestingly, the same three steroids were the more effective in increasing enzyme binding affinity to LUV membranes at the lower steroid–lipid molar ratio (Table 2; Fig. 4). Moreover, the docking scores for these three steroids matched the experimentally calculated binding affinities of rFAAH to membranes, pregnenolone being the most favored compound.

**Table 2.** Energetic Parameters for the Best Binding Modes of Steroids in the Membrane-Exposed Pocket of 2VYA.pdb Crystal Structure

	Cluster	ΔG (Kcal/mol)	Fullfitness (Kcal/mol)
Pregnenolone	1	−6.75	−1855
Progesterone	—		
Hydrocortisone	14	−6.52	−1816
Testosterone	—		
Cortisone	—		
Estradiol	5	−6.47	−1859

## Discussion

In this study, we show that all tested steroids seem to increase the binding of rFAAH to model membranes, but some of them (hydrocortisone and estradiol) exert their effect at a lower lipid–steroid molar ratio, whereas others (testosterone and cortisone) do so at a higher ratio probably by modifying the physicochemical properties of the membrane.

Pregnenolone was the molecule that most significantly raised rFAAH membrane affinity at both lipid–steroid ratios. It should be noted that the calculated membrane affinity value of rFAAH for pregnenolone-containing membranes (L_1/2_=21±2 μM) is almost as high as the value previously calculated for model membranes for which the enzyme has a very high affinity, that is, LUVs containing cholesterol and the FAAH substrate AEA (L_1/2_=15±7 μM) or ER membranes (L_1/2_=18±3 μM).^15^

Of note, pregnenolone has been shown to modulate eCB signalling also at the CB_1_ receptor level. Indeed, it acts as a negative modulator of CB_1_ by binding to an allosteric site.^26^

Docking analysis of putative binding sites for steroids on the surface of available crystal structures of rFAAH revealed a possible binding pocket in the hydrophobic patch of the protein, embedded in the lipid bilayer, that is suggestive of a direct protein–steroid interaction at the membrane interface that does not involve active site residues.

Thus, it can be speculated that steroids able to bind to this region of rFAAH can induce a protein conformational change that improves membrane binding affinity. In addition, binding of steroids to this region of rFAAH could also increase the hydrophobic surface of the protein that is involved in the interaction with LUVs.

An extension of this study by site-directed mutagenesis to further investigate the molecular details of steroid–FAAH interactions could help to identify the residues directly implicated in the binding.

In conclusion, this study shows for the first time that the interplay between eCBs and steroids could occur not only at the level of genomic regulation of ECS elements or of eCB-binding receptors, but also through a direct interaction of steroids with enzymes such as FAAH. The impact on FAAH catalytic activity remains to be ascertained in an independent study.

## Materials and Methods

All reagents used in this work were of the highest analytical grade. [^3^H]-AEA used for activity assay was purchased from Larodan Fine Chemicals AB (Malmo, Sweden). The LiposoFast apparatus for liposome formation was purchased from Avestin (Ottawa, Canada). pTrcHisA-FAAH-ΔTM was kindly provided by Prof. Benjamin F. Cravatt (Cellular Biology Department, University of San Diego, La Jolla, California). All other reagents were from Merck KGaA (Darmstad, Germany).

### Protein purification

rFAAH lacking a transmembrane domain (ΔTM) was purified from *Escherichia coli* BL21 (DE3) pLysS competent cells (Merck) as already described,^27^ using the pTrcHisAFAAH-ΔTM plasmid as reported^28^ and cloning for a His-tag enzyme lacking the N-terminal 29 residues sequence.

Before FRET measurements, enzymatic activity of rFAAH was assayed by measuring release of [^3^H]-ethanolamine from [^3^H]-AEA (60 Ci/mM) using liquid scintillation counting.^29^

### FRET measurements

The general protocol and FRET method for the investigation of the interaction of FAAH with model membranes can be found in Ref.^30^

In brief, synthetic POPC membranes at a lipid concentration of 2 mM in 50 mM Tris-HCl buffer, pH 7.5, were prepared in the absence and in the presence of the 1,2 dioleoyl-*sn*-glycero-3-phosphoethanolamine-*N*-(1-pyrenesulfonyl) (PyPE) fluorophore in a lipid–fluorophore molar ratio 200/1. POPC LUVs homogeneous in size with an average diameter of 100 nm were obtained by the extrusion method, using the LiposoFast™ extruder. Subsequently, steroids were added to liposome solutions at two different POPC–steroid molar ratios (30,000/1 and 300/1), and were incubated for at least 30 min at room temperature. Then, protein solutions dissolved in the same buffer were incubated with LUVs before FRET measurement, as previously described.^30^

FRET measurements were performed in a LS50b spectrofluorimeter (Perkin Elmer Inc., Waltham, MA), using as energy donors the protein tryptophan residues and as acceptors the PyPE fluorophore embedded in the LUVs. Binding isotherms were built and the membrane affinity of rFAAH for LUVs in all the considered experimental conditions was determined through nonlinear regression analysis of the fluorescence emission intensity as a function of lipid concentration [L], by determining the [L]_1/2_ value, that is, the concentration of lipid vesicles at half saturation of binding isotherm.^31^

FRET data points were collected in a POPC concentration range from 0 to 306 μM. Protein concentrations in Tris-HCl 50 mM buffer pH 7.5 ranged from 0.2 to 1.3 μM (see Ref.^30^).

The results presented for FRET analysis are means±standard deviation for at least three independent determinations. Statistical data analysis was performed with Prism 5 (GraphPad Software, Inc., La Jolla, CA), using the nonparametric one-way analysis of variance and the Bonferroni post-test.

### Docking analysis

The docking analysis was performed with the online web service Swissdock based on the EADock DSS (Dihedral Space Sampling) docking algorithm,^32^ according to which the prediction of the possible BMs of a small molecule with a target protein is based on the CHARMM^33^ set of force fields. BMs for ligands in cavities representing potential binding pockets of the protein are clustered and ranked according to the binding energy (ΔG) and the fullfitness value, an energetic parameter minimizing the target–ligand complex stability. The three-dimensional (3D) coordinates of pregnenolone were retrieved from the ZINC database.^34^ The coordinates for the target protein correspond to the monomeric structural unit of rat FAAH ΔTM 4DO3.pdb,^14^ 3QK5.pdb^23^ and 2VYA.pdb.^24^

Visualization and analysis of the docking results and related data were performed by means of UCSF Chimera.^35^ Protein 3D structure visualization and hydrophobic patches identification were performed with SPDBViewer.^36^

The 3D structures of the steroid molecules used in this study were retrieved from the Pubchem database.^37^

## Abbreviations Used

2-AG: 2-arachidonoylglycerol
3D: three-dimensional
AEA: anandamide (*N*-arachidonoylethanolamine)
AS: amidase signature
BMs: binding modes
CB_1_: type-1 cannabinoid receptor
CB_2_: type-2 cannabinoid receptor
CYP450: cytochrome P450
DSS: Dihedral Space Sampling
eCBs: endocannabinoids
ECS: endocannabinoid system
ER: endoplasmic reticulum
FAAH: fatty acid amide hydrolase
FRET: fluorescence resonance energy transfer
LUVs: large unilamellar vesicles
MA: membrane access
NSAID: nonsteroidal anti-inflammatory drug
POPC: 1-palmitoyl-2-oleoyl-*sn*-glycero-3-phosphocholine
PyPE: 1,2 dioleoyl-*sn*-glycero-3-phosphoethanolamine-*N*-(1-pyrenesulfonyl)
rFAAH: recombinant rat FAAH

## References

[1] Maccarrone M, Dainese E, Oddi S Intracellular trafficking of anandamide: new concepts for signaling. Trends Biochem Sci. 2010;35:601–608 20570522 10.1016/j.tibs.2010.05.008

[2] DiPatrizio NV, Piomelli D The thrifty lipids: endocannabinoids and the neural control of energy conservation. Trends Neurosci. 2012;35:403–411 22622030 10.1016/j.tins.2012.04.006 PMC3744874

[3] Silvestri C, Di Marzo V The endocannabinoid system in energy homeostasis and the etiopathology of metabolic disorders. Cell Metab. 2013;17:475–490 23562074 10.1016/j.cmet.2013.03.001

[4] Galve-Roperh I, Chiurchiù V, Díaz-Alonso J, et al. Cannabinoid receptor signaling in progenitor/stem cell proliferation and differentiation. Prog Lipid Res. 2013;52:633–650 24076098 10.1016/j.plipres.2013.05.004

[5] Maccarrone M, Guzmán M, Mackie K, et al. Programming of neural cells by (endo)cannabinoids: from physiological rules to emerging therapies. Nat Rev Neurosci. 2014;15:786–801 25409697 10.1038/nrn3846 PMC4765324

[6] Maccarrone M, Bab I, Bíró T, et al. Endocannabinoid signaling at the periphery: 50 years after THC. Trends Pharmacol Sci. 2015;36:277–296 25796370 10.1016/j.tips.2015.02.008 PMC4420685

[7] Pertwee RG Receptors and channels targeted by synthetic cannabinoid receptor agonists and antagonists. Curr Med Chem. 2010;17:1360–1381 20166927 10.2174/092986710790980050 PMC3013229

[8] Di Marzo V, De Petrocellis L Endocannabinoids as regulators of transient receptor potential (TRP) channels: a further opportunity to develop new endocannabinoid-based therapeutic drugs. Curr Med Chem. 2010;17:1430–1449 20166923 10.2174/092986710790980078

[9] Gasperi V, Dainese E, Oddi S, et al. GPR55 and its interaction with membrane lipids: comparison with other endocannabinoid-binding receptors. Curr Med Chem. 2013;20:64–78 23151004

[10] Rahman IAS, Tsuboi K, Uyama T, et al. New players in the fatty acyl ethanolamide metabolism. Pharmacol Res. 2014;86:1–10 24747663 10.1016/j.phrs.2014.04.001

[11] Di Marzo V, Maccarrone M FAAH and anandamide: is 2-AG really the odd one out? Trends Pharmacol Sci. 2008;29:229–233 18394720 10.1016/j.tips.2008.03.001

[12] Valiña ALB, Mazumder-Shivakumar D, Bruice TC Probing the Ser–Ser–Lys catalytic triad mechanism of peptide amidase: computational studies of the ground state, transition state, and intermediate. Biochemistry. 2004;43:15657–15672 15595822 10.1021/bi049025r

[13] Bracey MH, Hanson MA, Masuda KR, et al. Structural adaptations in a membrane enzyme that terminates endocannabinoid signaling. Science. 2002;298:1793–1796 12459591 10.1126/science.1076535

[14] Bertolacci L, Romeo E, Veronesi M, et al. A binding site for nonsteroidal anti-inflammatory drugs in fatty acid amide hydrolase. J Am Chem Soc. 2013;135:22–25 23240907 10.1021/ja308733u PMC3562592

[15] Dainese E, De Fabritiis G, Sabatucci A, et al. Membrane lipids are key modulators of the endocannabinoid-hydrolase FAAH. Biochem J. 2014;457:463–472 24215562 10.1042/BJ20130960

[16] Maccarrone M Central and peripheral interactions between endocannabinoids and steroids, and implications for drug dependence. Life Sci. 2005;77:1559–1568 15953622 10.1016/j.lfs.2005.05.006

[17] Miller WL Molecular biology of steroid hormone synthesis. Endocr Rev. 1988;9:295–318 3061784 10.1210/edrv-9-3-295

[18] Parker KL, Schimmer BP Transcriptional regulation of the genes encoding the cytochrome P-450 steroid hydroxylases. Vitam Horm. 1995;51:339–370 7483327 10.1016/s0083-6729(08)61044-4

[19] Hill MN, McEwen BS Endocannabinoids: the silent partner of glucocorticoids in the synapse. Proc Natl Acad Sci U S A. 2009;106:4579–4580 19293387 10.1073/pnas.0901519106 PMC2660761

[20] Ayakannu T, Taylor AH, Marczylo TH, et al. The endocannabinoid system and sex steroid hormone-dependent cancers. Int J Endocrinol. 2013;2013:259676 24369462 10.1155/2013/259676 PMC3863507

[21] Battista N, Pasquariello N, Di Tommaso M, et al. Interplay between endocannabinoids, steroids and cytokines in the control of human reproduction. J Neuroendocrinol. 2008;20(Suppl. 1):82–89 18426505 10.1111/j.1365-2826.2008.01684.x

[22] Karasu T, Marczylo TH, Maccarrone M, et al. The role of sex steroid hormones, cytokines and the endocannabinoid system in female fertility. Hum Reprod Update. 2011;17:347–361 21227997 10.1093/humupd/dmq058

[23] Gustin DJ, Ma Z, Min X, et al. Identification of potent, noncovalent fatty acid amide hydrolase (FAAH) inhibitors. Bioorg Med Chem Lett. 2011;21:2492–2496 21392988 10.1016/j.bmcl.2011.02.052

[24] Mileni M, Johnson DS, Wang Z, et al. Structure-guided inhibitor design for human FAAH by interspecies active site conversion. Proc Natl Acad Sci U S A. 2008;105:12820–12824 18753625 10.1073/pnas.0806121105 PMC2529035

[25] Palermo G, Bauer I, Campomanes P, et al. Keys to lipid selection in fatty acid amide hydrolase catalysis: structural flexibility, gating residues and multiple binding pockets. PLoS Comput Biol. 2015;11:e1004231 26111155 10.1371/journal.pcbi.1004231 PMC4481349

[26] Sabatucci A, Tortolani D, Dainese E, et al. *In silico* mapping of allosteric ligand binding sites in type-1 cannabinoid receptor. Biotechnol Appl Biochem. 2018;65:21–28 28833445 10.1002/bab.1589

[27] Di Venere A, Dainese E, Fezza F, et al. Rat and human fatty acid amide hydrolases: overt similarities and hidden differences. Biochim Biophys Acta. 2012;1821:1425–1433 22877990 10.1016/j.bbalip.2012.07.021

[28] Patricelli MP, Lashuel HA, Giang DK, et al. Comparative characterization of a wild type and transmembrane domain-deleted fatty acid amide hydrolase: identification of the transmembrane domain as a site for oligomerization. Biochemistry. 1998;37:15177–15187 9790682 10.1021/bi981733n

[29] Gattinoni S, De Simone C, Dallavalle S, et al. Enol carbamates as inhibitors of fatty acid amide hydrolase (FAAH) endowed with high selectivity for FAAH over the other targets of the endocannabinoid system. ChemMedChem. 2010;5:357–360 20112328 10.1002/cmdc.200900472

[30] Angelucci CB, Sabatucci A, Dainese E Measuring ECS interaction with biomembranes. Methods Mol Biol. 2016;1412:267–276 27245912 10.1007/978-1-4939-3539-0_27

[31] Qin S, Pande AH, Nemec KN, et al. The N-terminal α-helix of pancreatic phospholipase A2 determines productive-mode orientation of the enzyme at the membrane surface. J Mol Biol. 2004;344:71–89 15504403 10.1016/j.jmb.2004.09.034

[32] Grosdidier A, Zoete V, Michielin O SwissDock, a protein-small molecule docking web service based on EADock DSS. Nucleic Acids Res. 2011;39:W270–W277 21624888 10.1093/nar/gkr366 PMC3125772

[33] Brooks BR, Brooks CL, Mackerell AD, et al. CHARMM: the biomolecular simulation program. J Comput Chem. 2009;30:1545–1614 19444816 10.1002/jcc.21287 PMC2810661

[34] Irwin JJ, Shoichet BK ZINC—a free database of commercially available compounds for virtual screening. J Chem Inf Model. 2005;45:177–182 15667143 10.1021/ci049714 PMC1360656

[35] Pettersen EF, Goddard TD, Huang CC, et al. UCSF Chimera—a visualization system for exploratory research and analysis. J Comput Chem. 2004;25:1605–1612 15264254 10.1002/jcc.20084

[36] Guex N, Peitsch MC SWISS-MODEL and the Swiss-Pdb Viewer: an environment for comparative protein modeling. Electrophoresis. 1997;18:2714–2723 9504803 10.1002/elps.1150181505

[37] Kim S, Thiessen PA, Bolton EE, et al. PubChem substance and compound databases. Nucleic Acids Res. 2016;44:D1202–D1213 26400175 10.1093/nar/gkv951 PMC4702940

